# Serotonin and Interleukin 10 Can Influence the Blood and Urine Viscosity in Gestational Diabetes Mellitus and Pregnancy-Specific Urinary Incontinence

**DOI:** 10.3390/ijms242417125

**Published:** 2023-12-05

**Authors:** Danielle Cristina Honório França, Adenilda Cristina Honorio-França, Kênia Maria Rezende Silva, Fernanda Cristina Bérgamo Alves, Gabriela Bueno, Sarah Maria Barneze Costa, Aron Carlos de Melo Cotrim, Angélica Mércia Pascon Barbosa, Eduardo Luzía França, Marilza Vieira Cunha Rudge, The Diamater Study Group

**Affiliations:** 1 Department of Gynecology and Obstetrics, Botucatu Medical School, São Paulo State University, Botucatu 05508-070, SP, Brazil; daniellechfranca@gmail.com (D.C.H.F.); fernandacbalves15@gmail.com (F.C.B.A.); gabriela.bueno24@unesp.br (G.B.); sarah.barneze@unesp.br (S.M.B.C.); angelicapascon@gmail.com (A.M.P.B.);; 2 Biological and Health Sciences Institute, Federal University of Mato Grosso, Barra do Garças 78605-091, MT, Brazil; keniarezende1@gmail.com (K.M.R.S.); aroncarlosbg@gmail.com (A.C.d.M.C.); eduardo.franca@ufmt.br (E.L.F.); 3 Department of Physiotherapy and Occupational Therapy, School of Philosophy and Sciences, São Paulo State University, Marilia 17525-900, SP, Brazil

**Keywords:** hormone, immunomodulation, cytokines, rheology, involuntary loss of urine

## Abstract

Serotonin and interleukin 10 (IL-10) may play a role in gestational diabetes mellitus. Hyperglycemic environment, the detrusor musculature of the bladder and pelvic floor muscles may become damaged, leading to urination problems and urine viscosity in pregnant women with gestational diabetes mellitus and pregnancy-specific urinary incontinence. Urine and blood samples were collected from pregnant women between 24 and 28 weeks of gestation. The serotonin concentration and cytokine IL-10 levels were evaluated in plasma and urine. In the total blood and urine, the viscosity was evaluated in the presence and absence of exogenous serotonin and IL-10. The plasma serotonin levels decreased, while the urine serotonin levels increased in the normoglycemic incontinent (NG-I), hyperglycemic continent (GDM-C), and hyperglycemic incontinent (GDM-I) groups. The IL-10 in the plasma decreased in the GDM-I group and was higher in the urine in the NG-I and GDM-I groups. The blood viscosity was higher, independently of urinary incontinence, in the GDM groups. The serotonin increased the blood viscosity from women with GDM-C and urine in the NG-I, GDM-C, and GDM-I groups. Blood and urine in the presence of IL-10 showed a similar viscosity in all groups studied. Also, no difference was observed in the viscosity in either the blood or urine when in the presence of serotonin and IL-10. These findings suggest that serotonin and IL-10 have the potential to reduce blood viscosity in pregnant women with gestational diabetes and specific urinary incontinence, maintaining values similar to those in normoglycemic women’s blood.

## 1. Introduction

Gestational diabetes mellitus (GDM) is an important metabolic disease with increased insulin counter-regulatory hormones generating glucose intolerance, an inflammatory environment, abnormalities in oxidative stress, and altering the profile of released cytokines and other hormones [1,2].

In maternal hyperglycemia, the effects on cellular metabolism can affect systems and tissues, like the pelvic floor muscles (PFMs) and the urinary system, with pre-disposition to muscle weakening, atrophy, and pregnancy-specific urinary incontinence (PSUI) [3].

In the central nervous system, cytokines influence the urination pathways. In the peripheral nervous system, cytokines act on the sensitivity of the afferent nerve, the bladder, and the detrusor muscle tonus [3,4]. IL-10 (interleukin 10) is a cytokine with an autoregulatory and inhibitory role in producing inflammatory cytokines, promoting a suppressive effect on inflammatory processes [5]. This cytokine is present at lower concentrations in certain urogynecological pathologies [5] and pregnant women with gestational diabetes mellitus [6].

Cytokines can influence the central serotonergic pathways in the urinary system [7,8]. The serotonergic contraction of the bladder is altered in rats with an overactive bladder model [9]. Serotonergic paraneurons express serotonin, a hormone derived from tryptophan that can be associated with immunomodulatory responses, alterations in mechanisms of disorders, and the control of the appetite [3,7,10,11].

Research has indicated that serotonin may impact bladder activity in cases of pathological conditions, which is primarily influenced by inflammatory agents [12]. Urethral paraneurons produce and release serotonin, which is believed to be one of the hormones that can cause bladder contractions [13].

This hormone can regulate the production and release of pro-inflammatory cytokines, acting directly in the pathways responsible for changes in the maternal–fetal hyperglycemic environment [3]. Additionally, IL-10 can regulate serotonin transporter activity and serotonergic responses [14].

Dysfunction in the lower urinary tract is presented as the most common complication of diabetes mellitus, highlighting even more the interaction between GDM and PSUI [15]. The SLC6A4 gene, responsible for serotonin transportation, is linked to metabolic diseases, and gestational diabetes mellitus causes immunological changes in cytokine expression [1,16,17]. However, the influence of IL-10 and serotonin on pregnancy-specific urinary incontinence in pregnant women with gestational diabetes mellitus is not fully understood.

Endothelial dysfunction and vessel wall damage are common among diabetic patients [18]. Studies have found that hyperglycemia, cytokines, and serotonin can alter blood viscosity [19,20,21,22]. Serotonin has been found to reduce erythrocyte deformability and increase leukocyte adhesion, which can lead to sustained microcirculatory deficiencies and have a significant impact on the course of the disease [23,24]. The serotonergic pathway has been linked to urinary incontinence [25] as well as the development of gestational diabetes mellitus (GDM) [26]. Also, IL-10 appears to play an important role in this process by regulating serotonin activity and the production of serotonin reuptake receptors [14].

No study has yet linked hormonal factors and cytokines’ effects on blood viscosity in women with GDM and pregnancy-specific urinary incontinence. Thus, this study evaluated the influence of serotonin and IL-10 on blood and urine viscosity in pregnant women with gestational diabetes mellitus and pregnancy-specific urinary incontinence.

## 2. Results

The clinical information of the pregnant patients is presented in Table 1. Pregnant patients with diabetes independently of urinary incontinence were older than those in the NG-C and NG-I groups. In addition, the GDM-C and GDM-I groups had a higher percentage of overweight pregnant women and glycemic index (*p* < 0.05). However, no significant difference in urine density was observed between the groups (Table 1).

In women with gestational diabetes mellitus and urinary incontinence, there was a decrease in hemoglobin and hematocrit levels. The erythrocytes and lymphocytes increased, whereas the neutrophils decreased in the blood from women of the GDM-C group. No difference between the groups was observed in the leukocytes, monocytes, eosinophils, or platelets (Table 2).

**Figure 1:**
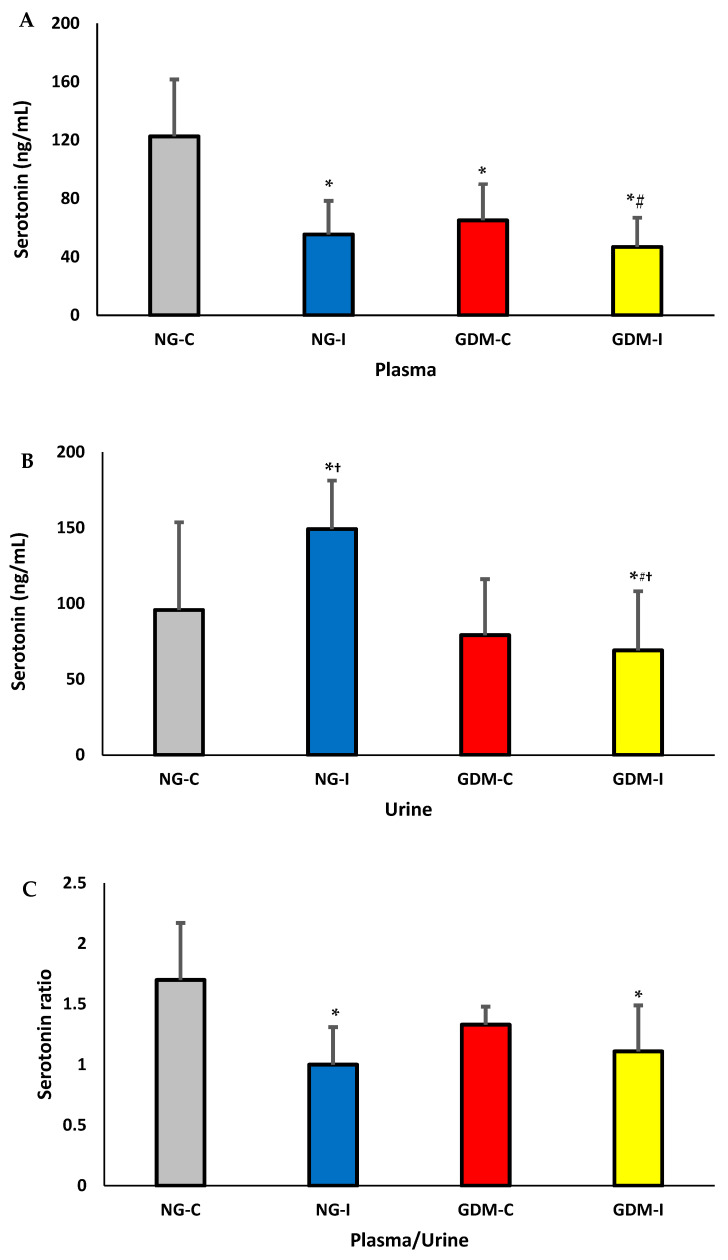
Serotonin levels in plasma (**A**), urine (**B**), and plasma/urine serotonin ratio (**C**) from pregnant patients with gestational diabetes mellitus (GDM) with or without urinary incontinence. NG-C: normoglycemic continent, NG-I: normoglycemic with urinary incontinence, GDM-C: gestational diabetes mellitus continent, and GDM-I: gestational diabetes mellitus with urinary incontinence. *p* < 0.05. * difference between the control group (NG-C) and the NG-I, GDM-C, and GDM-I groups; # difference between NG-I and GDM-I groups; † difference between plasma and urine, considering the same groups.

The serotonin levels in plasma and urine are shown in Figure 1. In the plasma, there was a decrease in the serotonin concentrations in the NG-I, GDM-C, and GDM-I groups (Figure 1A). The serotonin levels were higher in the urine in the NG-I group and lower in the GDM-I group. The highest serotonin levels in urine were observed in the NG-I group (Figure 1B). The plasma/urine serotonin ratio was lower in the NG-I and GDM-I groups (Figure 1C).

**Figure 2:**
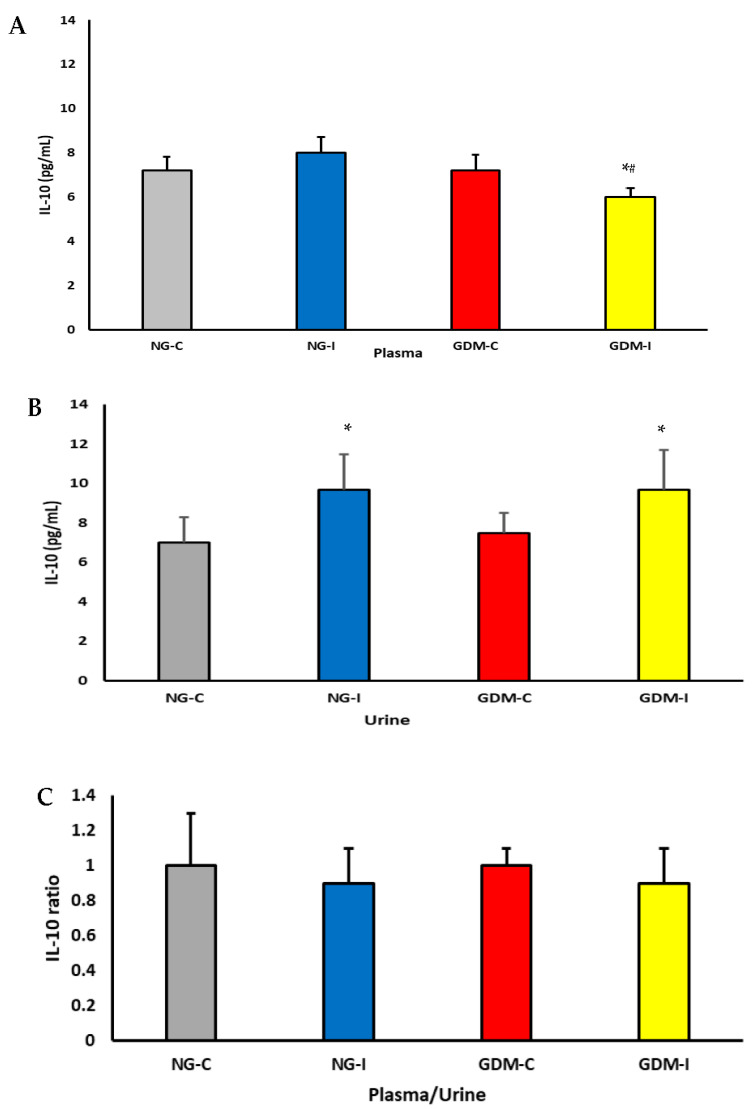
IL-10 cytokine levels (pg/mL) in plasma (**A**), urine (**B**), and plasma/urine serotonin ratio (**C**) from pregnant patients with gestational diabetes mellitus (GDM) with or without urinary incontinence. NG-C: normoglycemic continent, NG-I: normoglycemic with urinary incontinence, GDM-C: gestational diabetes mellitus continent, and GDM-I: gestational diabetes mellitus with urinary incontinence. *p* < 0.05. * difference between the control group (NG-C) and the NG-I, GDM-C, and GDM-I groups; # difference between NG-I and GDM-I groups.

The IL-10 levels in the plasma and urine are shown in Figure 2. In the plasma, there was a decrease in the IL-10 concentrations in the GDM-I (Figure 1A). The Il-10 levels were higher in the urine in the NG-I and GDM-I groups (Figure 1B). No difference was observed in the plasma/urine IL-10 ratio in the groups studied (Figure 1C).

The Pearson test correlated the serotonin and Il-10 levels in the plasma and urine (Table 3). The GDM-C group, in particular, exhibited a negative correlation of serotonin levels between plasma and urine. No correlation (*p* > 0.05) was found with serotonin levels in the NG-C, NG-I, or GDM-I groups. The IL-10 levels showed a negative correlation between plasma and urine in the NG-C group and a positive correlation in the NG-I group. No correlation (*p* > 0.05) was found with IL-10 levels in the hyperglycemic groups. There was a positive correlation between serotonin and IL-10 in the urine of the pregnant patients in the GDM-I group. No correlation (*p* > 0.05) was found between the serotonin and IL-10 levels in the plasma (Table 3).

**Figure 3:**
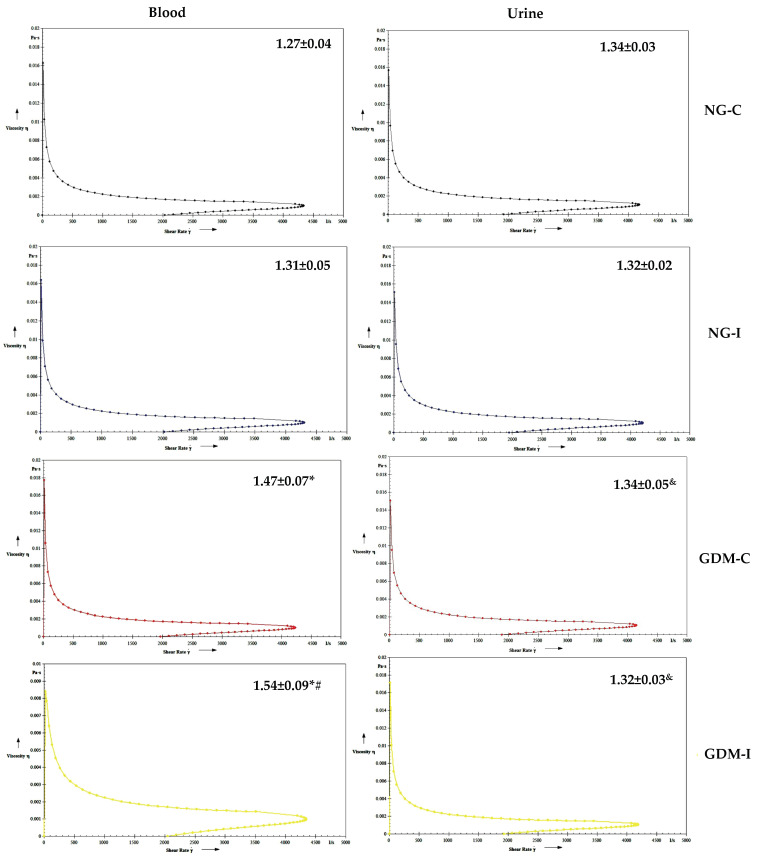
Blood and urine viscosity (mPa) in the groups. NGC: normoglycemic continent; NGI: normoglycemic incontinent; GDM-C: gestational diabetes mellitus continent; GDM-I: gestational diabetes mellitus incontinent. * difference between the control group (NG-C) and the NG-I, GDM-C, and GDM-I groups; # difference between NG-I and GDM-I groups; & difference between plasma and urine, considering the same groups.

Figure 3 shows the blood and urine viscosity. The blood viscosity was higher in the GDM-C and GDM-I groups compared to the NG groups (Figure 3). No difference was observed in the urine viscosity between the groups.

The Pearson test correlated the serotonin, viscosity, and IL-10 and serotonin in blood and urine (Table 4). The GDM-I group, in particular, exhibited a negative correlation between serotonin levels and viscosity in the blood. IL-10 correlated inversely with viscosity in the blood of pregnant NG-I and GDM-I patients. In urine, the serotonin positively correlated with viscosity in the GDM-C group. No correlation (*p* > 0.05) was found with IL-10 levels and viscosity in urine (Table 3).

**Figure 4:**
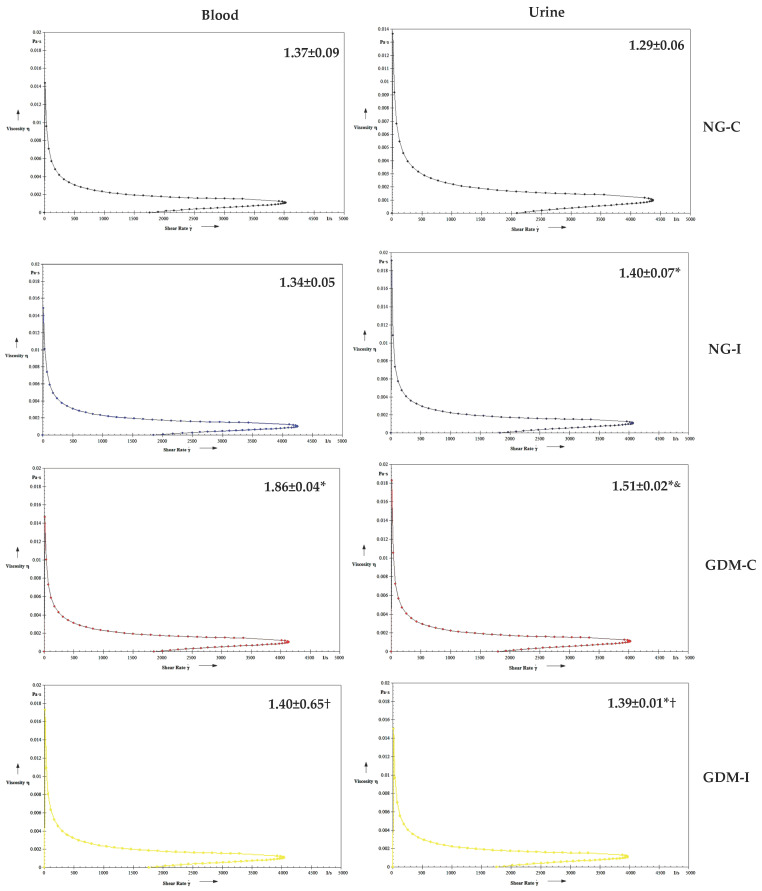
Blood and urine viscosity (mPa) treated with serotonin (100 ng/mL) in the groups. NGC: normoglycemic continent; NGI: normoglycemic incontinent; GDM-C: gestational diabetes mellitus continent; GDM-I: gestational diabetes mellitus incontinent. * difference between the control group (NG-C) and the NG-I, GDM-C, and GDM-I groups; † difference between GDM-C and GDM-I groups, & difference between plasma and urine, considering the same groups.

**Figure 5:**
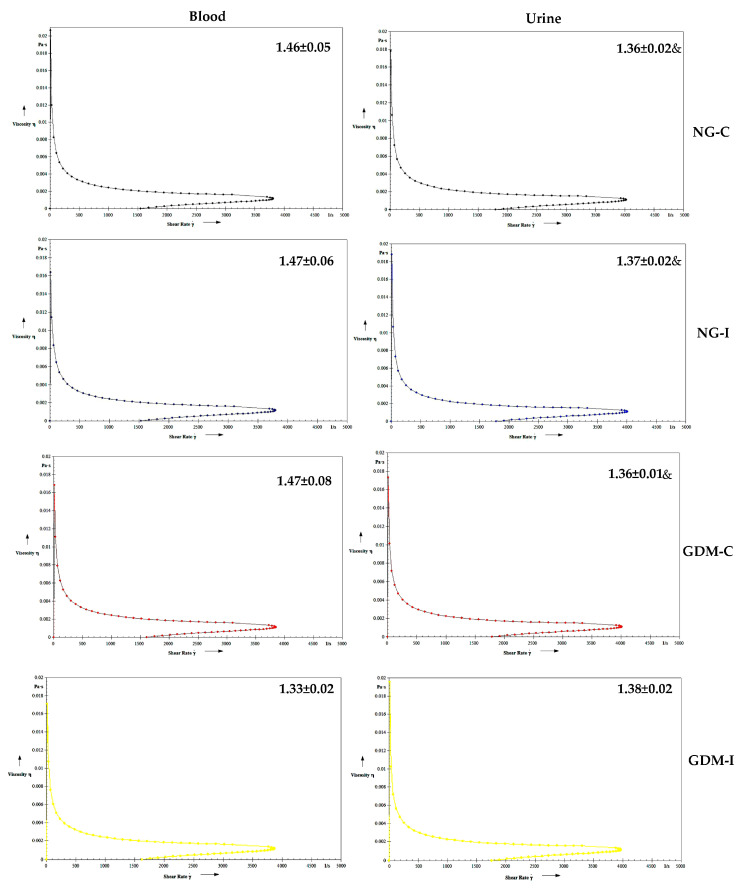
Blood and urine viscosity (mPa) treated with IL-10 cytokines (100 ng/mL) in the groups. NGC: normoglycemic continent; NGI: normoglycemic incontinent; GDM-C: gestational diabetes mellitus continent; GDM-I: gestational diabetes mellitus incontinent. & difference between plasma and urine, considering the same groups.

**Figure 6:**
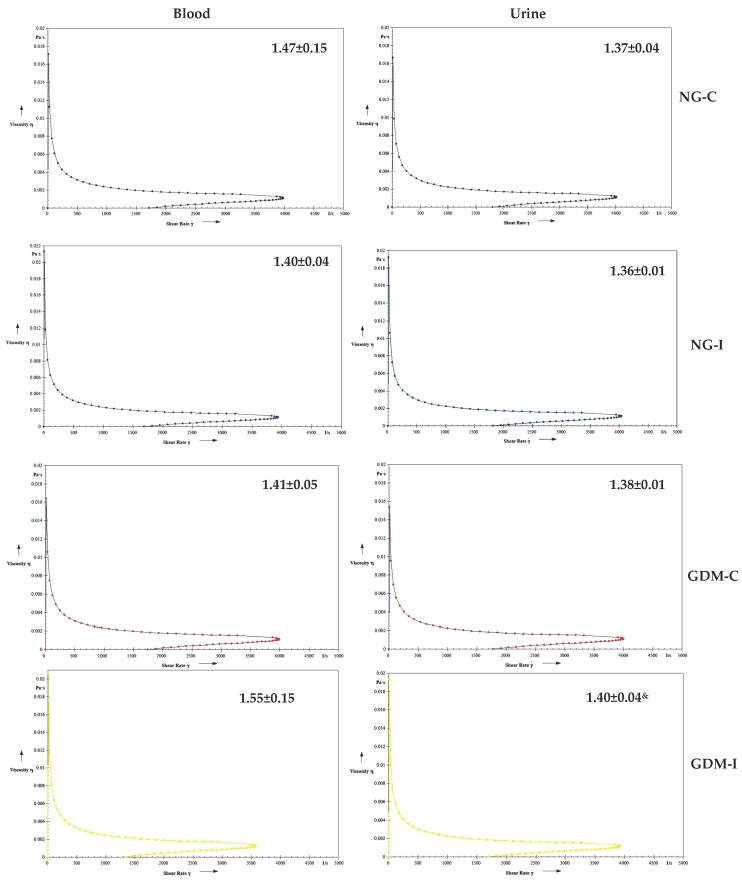
Blood and urine viscosity (mPa) treated with serotonin and IL-10 cytokines (100 ng/mL) in the groups. NGC: normoglycemic continent; NGI: normoglycemic incontinent; GDM-C: gestational diabetes mellitus continent; GDM-I: gestational diabetes mellitus incontinent. & difference between plasma and urine, considering the same groups.

The serotonin increased the blood viscosity in the blood of women with GDM-C and urine in the NG-I, GDM-C, and GDM-I groups (Figure 4). Blood and urine in the presence of IL-10 showed a similar viscosity in all groups studied (Figure 5). Also, no difference was observed in the viscosity in either blood or urine when in the presence of serotonin and IL-10 (Figure 6).

The viscosity showed a positive correlation with hematocrit (r = 0.7464; *p* = 0.0337) and a negative correlation with plaquettes (r = −0.8599; *p* = 0.0062) in the NG-I group. No correlation (*p* > 0.05) was found with viscosity or other blood parameters (Table 4).

## 3. Discussion

Diabetes can impact various systems in the body, such as the nervous and immune systems. In pregnant women with gestational diabetes mellitus, hyperglycemia can cause changes in cellular metabolism, muscle alterations in the pelvic and abdominal regions, and pregnancy-specific urinary incontinence [27,28]. In this work, women with GDM and PSUI showed a reduction in hemoglobin, hematocrit (Table 2), serotonin hormone (Figure 1), and anti-inflammatory cytokine interleukin 10 (IL-10) (Figure 2), and these alterations were reflected in blood viscosity (Figure 3).

Various risk factors can influence hyperglycemia in gestation, including advanced maternal age and increased body mass index (BMI). The risk of developing gestational diabetes mellitus (GDM) is higher in pregnant women with a higher BMI, which may be related to changes in their lipid metabolism [29,30]. In this study, women with gestational diabetes mellitus had higher glucose levels, regardless of urinary incontinence.

Diabetes can affect the entire body, causing micro and macro blood circulation changes. Pregnant women with gestational diabetes mellitus may experience hematological alterations [31]. Here, hyperglycemia is associated with an increase in erythrocytes and lymphocytes. Studies have shown that increased hemoglobin, platelets, and lymphocytes are linked to a higher risk of developing gestational diabetes mellitus (GDM) [32]. These changes in hematological parameters can lead to a chronic inflammatory environment, which can cause insulin resistance, impair the function of β cells, and affect insulin [32,33]. Interestingly, women with GDM and urinary incontinence present a decrease in hemoglobin and hematocrit levels, suggesting that the reduction in hematological parameters in women with GDM-I may be due to hyperglycemia associated with the involuntary loss of urine.

During pregnancy, hormonal factors are crucial, particularly when linked to hyperglycemia. Cystometric changes alter the neuromuscular function and smooth muscle cell action [34]. Serotonin receptor agonists have been trained in treating lower urinary tract dysfunctions associated with diabetes, and studies in diabetic rats show improved voiding efficiency and sphincter activity, pointing out that serotonin may have protective effects on smooth muscle disorders [35].

Studies conducted on pregnant women with gestational diabetes mellitus have shown decreased hormone serotonin [36]. In this study, regardless of urinary incontinence, there was a decrease in the serotonin concentrations in plasma and urine in women with GDM. However, there were lower serotonin levels in the plasma in the group of normoglycemic pregnant women who also had urinary incontinence. At the same time, the concentration of this hormone in the urine was high. The lower blood/urine ratio of the hormone confirmed this in both groups with urinary incontinence. These findings support the notion that hyperglycemia associated with involuntary urine loss can affect serotonergic pathways.

Urinary incontinence can be influenced by genetic factors and the serotonergic pathway [25]. Neurochemistry studies reveal that sphincter motor neurons have high concentrations of neurotransmitters and receptors [37]. The regulation of bladder function is influenced by central serotonergic modulation, and neurotransmitter changes can affect urination and may explain the association with urinary incontinence [38]. Reduced serotonin function in the brain and spinal cord can lead to a decrease in bladder activity [37]. Research on rodents has shown that decreasing serotonin in the brain can cause changes in urinary frequency, which can be corrected with serotonin reuptake inhibitors [39]. This provides further evidence that abnormal serotonin functions can be associated with urinary incontinence [40].

Serotonergic modulation is essential in controlling continence by acting along the neural, medullary, and supramedullary routes. It also affects the central serotonergic pathways in the tissue environment of the detrusor smooth muscle, the skeletal muscle of the external urethral sphincter, and the pelvic floor [41]. Changes in serotonin excretion and reduced plasma serotonin levels can lead to glucose intolerance and increase the risk of gestational diabetes [42]. Here, in women with gestational diabetes, the serotonin levels in the plasma are inversely proportional to those found in urine. It is noteworthy that serotonin signaling in pancreatic islets plays a crucial role during pregnancy [43].

There is a lack of information on serotonin metabolism in mothers with GDM due to the complex nature of this process and its relevance to glucose regulation during pregnancy [26]. Serotonin is mainly known for its effects on the central nervous system and critical role in regulating physiological processes [44]. It is co-secreted with insulin in a glucose-dependent manner [45] and is involved in physiological pathways and adaptive processes during pregnancy [46]. Research using metabolic markers in urine and plasma has demonstrated that serotonin plays a role in the onset of GDM [26,47] and that the serotonergic pathway contributes to urinary incontinence [25], which can lead to a homeostatic imbalance. Therefore, maintaining physiological levels of both serotonin and IL-10 is crucial in women with GDM urinary incontinence.

Gestational diabetes mellitus is characterized by an inflammatory state in which cytokines play an important role in the genesis and control of inflammation processes [48]. Insulin resistance can also result from the cytokines, which may be the molecular basis of the inflammation in the disease [49]. Serotonin can stimulate the release and production of pro-inflammatory cytokines, but the essential anti-inflammatory cytokine, IL-10, can modulate serotonin by regulating the activity of serotonin transporters, the production of serotonin reuptake receptors, and serotonergic responses [14].

Maintaining a balance between pro-inflammatory and anti-inflammatory cytokines is crucial for a successful pregnancy, and the immune system plays a significant role in this process [1,50]. During pregnancy, an anti-inflammatory status dominates, which is essential for the natural progression of pregnancy, especially in the case of diabetes [51,52]. However, certain factors, like metabolic diseases, may predispose individuals to decreased levels of anti-inflammatory cytokines [51]. A lower level of the anti-inflammatory IL-10 in the blood has been linked to insulin resistance, metabolic syndrome, and type II diabetes [53]. Pregnancy can affect the immune system [52], so decreasing anti-inflammatory factors could result in disorders such as GDM.

The current study has demonstrated that the cytokine IL-10 was reduced in plasma and increased the excretion in the urine of pregnant women with gestational diabetes and urinary incontinence. Interestingly, the IL-10 levels increased in the urine of pregnant women with normoglycemic urinary incontinence and showed a positive correlation between plasma and urine in this group and a positive correlation between serotonin and IL-10 in the urine of pregnant patients with gestational diabetes and urinary incontinence. These findings indicate that hyperglycemia, especially when combined with involuntary loss of urine, can decrease the production of IL-10 in the body. This decrease in IL-10 levels may be associated with reduced serotonin levels and greater excretion of the cytokines in the urine, leading to an imbalance in anti-inflammatory mechanisms and promoting inflammation.

The pathogenesis of gestational diabetes may be linked to the hematological changes and increased viscosity caused by chronic inflammation. Abnormalities in hematological cells may reflect maternal immune dysregulation and viscosity, playing a role in GDM pathophysiology [32]. In this study, the blood viscosity increased in both gestational diabetes groups (GDM-C and GDM-I), suggesting that hormones and cytokines may affect blood rheological parameters in patients with gestational diabetes and urinary incontinence.

Rheological parameters are used in cardiovascular research to detect patients prone to cardiovascular disease and to monitor women at risk of pre-eclampsia [54]. The typical inflammatory process of diabetes can alter blood flow, which may cause microcirculation and blood viscosity changes, which are crucial for maintaining the structural integrity and non-deformation of cells [55].

Studies have reported changes in the structure of red blood cells and increased blood viscosity in diabetic patients [20]. Furthermore, the use of immunomodulatory agents has been linked to the recovery of cell structure and function [21,50] and an improvement in blood viscosity [20], which may lead to better flow characteristics.

The use of immunomodulators during pregnancy has gained global interest. Current evidence mainly reports outcomes related to the newborn and the potential effect on the ontogeny of the immune system [56]. Previous studies have reported the effects of cytokines [21,57] as immunomodulators capable of acting on blood viscosity. However, this study is the first to describe changes in viscosity and the potential use of immunomodulators to restore viscosity in pregnant women with GDM and urinary incontinence. Yet there remains a dilemma regarding prioritizing the risk of continuing the medication during pregnancy to the detriment of the risk of maternal disease action.

In some cases, there is a recommendation to interrupt biological therapies at the beginning of pregnancy, except in cases where the potential benefits for the mother outweigh the potential risk for the fetus [58]. In this sense, future research should address gaps in the knowledge about the use of immunomodulators during pregnancy, especially in pregnant women with gestational diabetes and urinary incontinence. This would help to evaluate the impact of molecule biological therapies on fetal/newborn organogenesis and the effect of concomitant immunomodulators on maternal disease action [58,59].

Considering that serotonin and IL-10 levels are altered in pregnant women with gestational diabetes and urinary incontinence, and there was a positive correlation between viscosity and serotonin and between viscosity and IL-10 in the blood of these pregnant patients, we aimed to verify the possible effects of this hormone and cytokine on viscosity. The treatment with IL-10 or combined IL-10–serotonin was associated with reduced viscosity. The clinical potential for using cytokines [21,60,61] has been reported for various diseases.

The biological significance of differences in IL-10 and serotonin levels and the restoration of viscosity associated with two agents in the blood from mothers with gestational diabetes and urinary incontinence noted in our study indicates the possible use of this cytokine as a potential immunomodulatory agent. Further studies are needed to verify the mechanisms involved in changes in serotonin and IL-10 levels in blood and urine and the modulatory role of these bioactive components in gestational diabetes associated with pregnancy-related urinary incontinence.

It is important to note that the data collected in this study only pertain to a specific period and do not account for the severity or persistence of urinary incontinence during and after pregnancy. This limitation highlights the need for further investigation into the effects of gestational diabetes mellitus (GDM) on urinary continence, as well as the impact of other factors during pregnancy. Follow-up studies with pregnant women suffering from GDM are necessary to evaluate hyperglycemia and urinary incontinence with a focus on other contributing factors.

In summary, this study provides evidence of the complexity and relevance of neuroimmunoendocrine interactions in pregnant women. Metabolic changes and excess circulating glucose in pregnant women with gestational diabetes and urinary incontinence can lead to serious complications since pregnant women present changes in serotonin and IL-10 cytokine levels, as well as in hematological and rheological parameters, and monitoring and controlling glucose levels are crucial to preventing adverse outcomes. In gestational diabetes, increased blood viscosity can cause hypertensive peaks that disrupt fetal balance in the intrauterine cavity. High blood viscosity and hyperglycemia can also alter the fetoplacental unit, resulting in complications related to the loss of gestational balance [62,63,64]. Early diagnosis and treatment can prevent progression in patients with GDM-I. Assessing blood and urinary viscosity in pregnant patients can be an easy, quick, and economically viable means of early analysis of the association between GDM-I and high blood viscosity.

Furthermore, in the clinical monitoring of pregnant women postpartum, it can also be used to predict long-term urinary incontinence, evaluating at 6 and 12 months after birth. It would then be a viable means of monitoring these postpartum women. In this way, possible therapeutic interventions and monitoring in pregnancies planned using these parameters can be started early and after pregnancy.

## 4. Materials and Methods

### 4.1. Study Design and Participants

A cross-sectional study was conducted in the Perinatal Diabetes Research Center, Botucatu, São Paulo, Brazil. Out of the 114 pregnant patients who were examined, the inclusion criteria were being pregnant with a single live fetus, undergoing the oral glucose tolerance test between the 24th and 28th week of pregnancy, being at least 18 years old and at most 40 years old, signing the informed consent form, not taking antidepressants, and not having autoimmune diseases. In total, 17 pregnant patients with pre-existing conditions such as type 1 or type 2 diabetes, obesity, autoimmune diseases, fetal malformations, twin pregnancies, aged under 18 or over 40, or who used antidepressants were excluded from the study.

**Figure 7:**
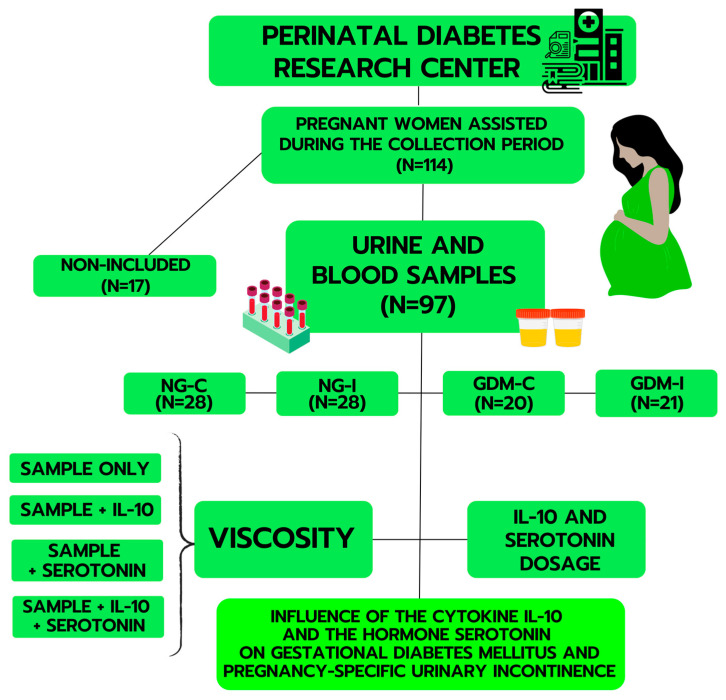
Representative scheme for obtaining samples and experimental design.

Fasting plasma glucose was determined in the first trimester of pregnancy. The pregnant women underwent a 75 g oral glucose tolerance test (OGTT-75g) between the 24th and 28th week of pregnancy. The OGTT-75g was altered if any of the following plasma glucose values were met or exceeded: fasting glycemia of 92 mg/dL, post-load 1 h glucose of 180 mg/dL, and post-load 2 h glucose of 153 mg/dL [65]. The pregnant women also completed the International Consultation on Incontinence Questionnaire Short Form (ICIQ-SF) [66], which is a validated tool that assesses the impact of urinary incontinence (UI) on a person’s quality of life. The questionnaire helps to classify urinary loss and the severity index of UI (ISI) [67] and is also validated as a quantitative and semi-objective measure of the severity of UI. Based on the results of the OGTT-75g and the ICIQ-SF questionnaire, the pregnant women were divided into four groups according to their blood glucose results during the oral glucose tolerance test and urinary continence: normoglycemic continent (NG-C), normoglycemic with pregnancy-specific urinary incontinence (NG-I), gestational diabetes mellitus continent (GDM-C), and gestational diabetes mellitus with pregnancy-specific urinary incontinence (GDM-I). Eligible participants voluntarily agreed to participate in the study. The sample consisted of 28 participants in group NG-C, 28 in group NG-I, 20 in group GDM-C, and 21 in group GDM-I, totaling 97 participants. The scheme for obtaining samples and experimental design is described in Figure 7. All patients signed the Informed Consent Form.

### 4.2. Blood and Plasma Separation

Blood samples were collected in tubes containing EDTA via venipuncture (approximately 5 mL) from pregnant women who attended the Perinatal Diabetes Research Central at the Botucatu Medical School (FMB-UNESP) during the project development period. A part of the blood samples was centrifuged at room temperature for 15 min at 160× *g* to obtain the plasma and stored at −80 °C for posterior IL-10 and serotonin assays. Another part of the total blood samples was reserved for the hematological and hemorheology analyses.

### 4.3. Urine Separation

The participants collected urine samples from the medium flow using specific urine collection bottles and following instructions for cleanliness. The samples were centrifuged at 160× *g* for 15 min, and then separated and stored at −80 °C for further analysis.

### 4.4. Plasma Glucose Determination

Fasting plasma glucose was determined in the first trimester of pregnancy. Samples of 20 μL plasma, the standard of 100 mg/dL (BioTécnica^®^, Ref 10.008.00, Varginha, Brazil), were placed in 2.0 mL phosphate-buffered solution (0.05 M, pH 7.45, with amino antipyrine 0.03 mM, 15 mM sodium p-hydroxybenzoate, 12 kU/L glucose oxidase and 0.8 kU/L peroxidase). The suspensions were mixed and incubated for 5 min at 37 °C. The reactions were read on a spectrophotometer at 510 nm.

### 4.5. Hematological Parameters

The total blood samples with EDTA from the pregnant women were sent for laboratory analysis to obtain hematological data such as erythrogram, leukogram, and platelet count using an automated device (Sysmex KX-21N™, Kōbe, Hyōgo, Japan) [68].

### 4.6. Serotonin Determination

The quantitative determination of serotonin hormone concentration in human plasma and urine was performed via enzyme immunoassay in ELISA microplates with the following characteristics: the lower detection limit was 3.97 ng/mL, and the intra-assay and inter-assay coefficients of variation (%) were >10% and >12%, respectively. According to the manufacturer’s instructions, the serotonin present in the plasma was determined using an ELISA kit from Cloud-Clone Corp. (Katy, TX, USA). The reaction values were measured by absorbance in a spectrophotometer with a 450 nm filter. The results were obtained through a standard curve and expressed in ng/mL.

### 4.7. IL-10 Cytokine Determination

The concentrations of cytokine IL-10 present in the serum and urine samples were evaluated using the “Cytometric Bead Array” Kit (CBA, BD Bioscience, San Jose, CA, USA). The bead with fluorescence intensity was conjugated with a specific capture antibody for cytokines, mixed to form the CBA, and read in the FL3 channel of the FACSCalibur flow cytometer (BD Biosciences^®^, San Jose, CA, USA). The bead populations were visualized according to their respective fluorescence intensities: from least bright to brightest. In the CBA, the cytokine capture beads were mixed with the detection antibody conjugated to the fluorochrome PE and then incubated with the samples to form the “sandwich” assay. The acquisition tubes were prepared with 50 μL of the sample, 50 μL of bead mixture, and 50 μL of detection reagent (PE Detection Reagent/1 vial, 4 mL). The same procedure was performed to obtain the standard curve. The tubes were incubated for three hours at room temperature without light. The cytokine was analyzed using flow cytometry (FACSCalibur, BD Bioscience, San Jose, CA, USA). The results were generated in graphs and tables using the CellQuest Pro (BD)^®^ software (BD Bioscience, San Jose, CA, USA), and the data were analyzed using the FCAP Array software v3.0 (BD Bioscience, San Jose, CA, USA).

### 4.8. Hemorheological Parameters

The total blood with EDTA from each woman was subjected to hemorheology analysis to obtain data on blood viscosity. A compact modular rheometer, Anton-Paar^®^ Cone-Plate model—MCR 102 (Anton Paar^®^ GmbH, Ostfildern, Germany), was used, and the graphs were obtained using the Rheoplus software v3.6.1 (Anton Paar, Ostfldern, Germany). For the hemorheology analysis, 750 μL of whole blood with EDTA was used, maintaining a temperature of 37 °C in the plate with 60 flow measurement points [20].

### 4.9. Treatment of Blood with IL-10 and Serotonin

To assess the effect of IL-10 and serotonin on blood viscosity, a 580 μL sample of blood was incubated with 20 μL of IL-10 (Sigma, St Louis, MO, USA; final concentration 100 pg/mL) and/or serotonin (Sigma, St. Louis, MO, USA; final concentration 100 pg/mL) for 1 h at 37 °C. The dose–response curve previously determined this concentration. The blood was used immediately for the rheological analysis.

### 4.10. Statistics

The statistical analyses were performed using the BioEstat 5.0 program—qualitative variables through relative and absolute frequency. Quantitative variables were described using their means and standard deviation, and data were analyzed for normal distribution using the Shapiro–Wilk test. The ANOVA test was used to compare mean values; the Tukey post-test was used for further group analysis. For the correlation analysis, the Pearson test was used. The adopted significance criterion was 5% (*p* < 0.05).

## 5. Conclusions

Based on the data from the present study, it can be concluded that pregnant women with gestational diabetes mellitus and pregnancy-specific urinary incontinence experience decreased hemoglobin, hematocrit, serotonin, and IL-10 in their blood. These changes increase blood viscosity, suggesting an interaction between hematological, hormonal, endocrine, and immunological components during the disease. Hyperglycemia due to gestational diabetes has been associated with lower plasma serotonin and IL-10 levels. This research highlights the potential of serotonin and IL-10 in reducing the blood viscosity of pregnant women with gestational diabetes and pregnancy-specific urinary incontinence and maintaining values similar to those found in the blood of normoglycemic women.

## Figures and Tables

**Table 1:** Clinical data on pregnant women divided into four groups based on their glycemic levels and urinary continence: normoglycemic continent (NG-C), normoglycemic with pregnancy-specific urinary incontinence (NG-I), gestational diabetes mellitus continent (GDM-C), and gestational diabetes mellitus with pregnancy-specific urinary incontinence (GDM-I).

Groups	NG-C (N = 28)	NG-UI (N = 28)	GDM-C (N = 20)	GDM-I (N = 21)
Age	25.14 ± 4.68	26.53 ± 6.91	30.40 ± 6.42	30.47 ± 6.56
BMI categories (%):				
Normal weight	58.8	51.7	38.6	36.7
Overweight	41.2	48.3	61.4	63.3
Obesity	0	0	0	0
Glucose level (mg/dL)	75.28 ± 6.87	75.18 ± 7.37	92.12 ± 9.88 *	90.32 ± 12.18 *#
Urine Density	1.01 ± 0.011	1.01 ± 0.008	1.01 ± 0.006	1.01 ± 0.006

Note: BMI: body mass index categories (normal weight: 18.5 and 24.9 kg/m^2^, overweight: 25 and 29.9 kg/m^2^, and obesity: over 30 kg/m^2^); data correspond to the median and standard deviation of the pregnant women. *p* < 0.05. * difference between the control group (NG-C) and the NG-I, GDM-C, and GDM-I groups; # difference between NG-I and GDM-I groups.

**Table 2:** Hematological values in the blood from women were divided into groups based on their glycemic levels and urinary continence: normoglycemic continent (NG-C), normoglycemic with urinary incontinence (NG-I), gestational diabetes mellitus continent (GDM-C), and gestational diabetes mellitus with urinary incontinence (GDM-I).

Parameters	NG-C	NG-I	GDM-C	GDM-I	*p*
Erythrocytes (10^6^/μL)	4.10 ± 0.37	4.00 ± 0.41	4.37 ± 0.33 *	4.00 ± 0.43 #	0.0361
Hemoglobin (g/dL)	12.57 ± 0.88	12.37 ± 1.06	13.06 ± 1.04	11.91 ± 1.30 *#	0.0242
Hematocrit (%)	38.56 ± 2.32	38.18 ± 3.08	39.11 ± 2.91	35.87 ± 3.52 *#	0.0239
Leukocytes (10^3^/μL)	9.68 ± 2.15	9.26 ± 2.66	9.12 ± 1.66	10.12 ± 2.60	0.5677
Neutrophils (%)	69.33 ± 5.77	69.08 ± 5.54	57.56 ± 20.01 *	68.88 ± 7.17	0.0019
Lymphocytes (%)	21.30 ± 4.92	21.91 ± 4.39	31.63 ± 17.09 *	21.30 ± 5.12	0.0011
Monocytes (%)	7.48 ± 1.51	7.22 ± 1.55	8.56 ± 2.59	7.52 ± 2.50	0.1236
Eosinophils (%)	1.80 ± 0.96	1.84 ± 1.38	1.98 ± 1.12	2.17 ± 2.71	0.2317
Platelets (10^3^ μL)	251.32 ± 57.92	232.25 ± 60.00	284.84 ± 51.89	250.67 ± 73.97	0.1017

Data correspond to the mean and standard deviation of the pregnant women. *p* < 0.05. * difference between the control group (NG-C) and the NG-I, GDM-C, and GDM-I groups; # difference between GDM-C and GDM-I groups.

**Table 3:** Pearson’s linear correlations and values of linear coefficients (r) and statistical significance (* *p*) of serotonin (ng/mL) and IL-10 (pg/mL) between plasma and urine and between serotonin (pg/mL) and IL-10 (pg/mL) in the plasma and urine in the groups. NGC: normoglycemic continent; NGI: normoglycemic incontinent; GDM-C: gestational diabetes mellitus continent; GDM-I: gestational diabetes mellitus incontinent.

Groups	NG-C	NG-I	GDM-C	GDM-I
Plasma/Urine				
Serotonin	r = −0.1617 *p* = 0.7291	r = −0.4782 *p* = 0.2777	r = −0.8138 * *p* = 0.0487	r = 0.1442 *p* = 0.7578
IL-10	r = −0.7273 * *p* = 0.0408	r = 0.8413 * *p* = 0.0486	r = 0.3988 *p* = 0.3277	r = 0.2742 *p* = 0.5110
Serotonin/IL-10 cytokine				
Plasma	r = −0.4946 *p* = 0.2127	r = −0.2316 *p* = 0.5810	r = 0.4427 *p* = 0.2720	r = −0.1846 *p* = 0.6616
Urine	r = 0.1782 *p* = 0.6728	r = −0.3080 *p* = 0.4580	r = 0.0706 *p* = 0.8680	r = 0.9379 * *p* = 0.0331

**Table 4:** Pearson’s linear correlations and values of linear coefficients (r) and statistical significance (* *p*) of the viscosity (mPa) between serotonin (pg/mL) and IL-10 (pg/mL) in the blood and urine of women from the groups. NG-C: normoglycemic continent; NG-I: normoglycemic incontinent; GDM-C: gestational diabetes mellitus continent; GDM-I: gestational diabetes mellitus incontinent.

	Viscosity	NG-C	NG-I	GDM-C	GDM-I
Blood	Serotonin	r = 0.5933 *p* = 0.1210	r = −0.4010 *p* = 0.3248	r = −0.4620 *p* = 0.2490	r = −0.7082 * *p* = 0.0493
	IL-10	r = 0.4225 *p* = 0.2970	r = −0.7775 * *p* = 0.0231	r = 0.6091 *p* = 0.1089	r = −0.8069 * *p* = 0.0155
Urine	Serotonin	r = 0.3562 *p* = 0.3865	r = −0.0682 *p* = 0.8724	r = 0.7711 * *p* = 0.0250	r = −0.5800 *p* = 0.1317
	IL-10	r = 0.2602 *p* = 0.5337	r = 0.2505 *p* = 0.5496	r = −0.3784 *p* = 0.3553	r = −0.1908 *p* = 0.6513

## Data Availability

The authors will make the data supporting this study’s interpretations available if requested.
